# On Inflamed Nipples during Lactation

**Published:** 1858-11

**Authors:** A Lady


### *On Inflamed Nipples during Lactation.*

BY A LADY.

[It is not often that medical journals are so fortunate at to secure the coöperation of lady-contributors. Our readers will, however, recognize in the beneficent spirit and practical sense of the following communication the best characteristics, moral and intellectual, of the *belle sex.* Far be it from us to use the privilege accorded us of *fixing up* the article : no editor of the coarser sex could have expressed what our fair contributor has said, with one tithe of her own appropriate diction. The letter is addressed to our friend and colleague, Prof. Maddin : we envy him his correspondent. It will be seen that we are to be favored with some articles from the lady's husband, an eminent practical physicians. We can only say, that if he is half as good a writer as his lady, we wish to see his contributions in every number.

Our friend and brother editor, Dr. Huston, as will be seen, is deprived of a contributor by our gladly accepting the paper. Forgive us, brother H., and copy the article: it is well worthy of a place in your excellent family journal.—Ed.]

Dear Doctor :—You wrote to my husband, requesting him to send you an article for your journal. He wishes me to say that his time is so entirely occupied with his practice, you must excuse him for the present; but when he has more leisure, you shall hear from him. Would you not like for me to write you a paper—something that will make every mother in the land bless you ? I am now nursing my sixth child, and have suffered very much with all of them from that worst of all troubles. While nursing A——, the youngest child but one, I had sore nipples for eight months, and tried all the

*doctor*-remedies, but found no relief—yes, and every thing any one would tell me, until I had exhausted the whole vocabulary of remedies *then known;* but still no relief. When my last babe was born, I thought, surely I will avoid this worst of annoyances; for I had tried to prepare my nipples by using brandy and alum solution, together with other washes, to harden the nipples; but all to no purpose; for the first time my infant was put to the breast, they were worse, as I thought, than they had ever been—all cracked open and bleeding. Now for the remedy:

Gum Arabic, dissolved in boiling-hot water: make a *very thick* mucilage, as much as can be dissolved in a small quantity of water. I put up a vial-full of this, and every time after I would nurse my babe, I would put it upon the nipples with a feather or camel's-hair pencil, so tender were my breasts, and then place a piece of soft linen over them. I never had such relief in my life. Every preparation I had ever used before, would cause as great pain as if a live coal of fire had been placed upon them; but this was cooling, soothing, and allayed the inflammation, and the chaps and gashes all healed rapidly. I then tried the gum Arabic in powder, and found it to produce the same effect. I must not omit to say that the nipple should be well moistened before giving it to the child, and then use the mucilage immediately, every time it nurses.

Now, Doctor, I feel that I wish every mother to be in possession of this simple but efficient remedy. Not the least of its recommendations is, that it is entirely harmless to the child. If you choose you may have it to put in your journal after fixing it up as it ought be written. In my opinion this fact is worth fifty pages about your medical reforms, and a thousand pages of your quarrelling discussions, about who shall be the next President of the American Medical Association. You may laugh at this; but if you don't publish the remedy, I will send it to Dr. Huston for the Home Circle.

Respectfully your friend, etc.

[*Nashville Record of Med. & Phy. Science.*]

